# Computed Tomography Angiography of Carotid Arteries and Vertebrobasilar System: A Simulation Study for Radiation Dose Reduction

**DOI:** 10.1097/MD.0000000000001058

**Published:** 2015-07-02

**Authors:** Manuel Kramer, Stephan Ellmann, Thomas Allmendinger, Achim Eller, Ferdinand Kammerer, Matthias S. May, João F. Baigger, Michael Uder, Michael M. Lell

**Affiliations:** From the Institute of Radiology (MK, SE, AE, FK, MSM, JFB, MU, MML), University of Erlangen-Nuremberg, Maximiliansplatz 1, Erlangen, Germany; and Siemens Healthcare (TA), Forchheim, Germany.; ∗ MK and SE contributed equally to this work.

## Abstract

Computed tomography angiography (CTA) of carotid arteries and vertebrobasilar system is a standardized procedure with excellent image quality, but radiation exposure remains a matter of concern. The aim of this study is to examine to what extent radiation dose can be lowered in relation to a standard protocol by simulating examinations with lower tube currents applying a dedicated software.

Lower tube current was simulated by a dedicated noise insertion and reconstruction software (ReconCT). In a phantom study, true scans were performed with different dose protocols and compared to the results of simulated dose reductions of the same degree, respectively. In a patient study, 30 CTAs of supra-aortic vessels were reconstructed at a level of 100%, 75%, 50%, and 25% of the initial dose. Objective and subjective image analyses were performed.

No significant noise differences between true scans and simulated scans of mimicked contrasted vessels were found. In the patient study, the quality scores of the 4 dose groups differed statistically significant; this difference vanished for the comparison of the 100% and 75% datasets after dichotomization into the categories of diagnostic and nondiagnostic image quality (*P* = .50).

This study suggests an easy-to-implement method of simulating CTAs of carotid arteries and vertebrobasilar system with lower tube current for dose reduction by artificially adding noise to the original raw data. Lowering the radiation dose in a moderate extent to 75% of the original dose levels does not significantly alter the diagnostic image quality.

## INTRODUCTION

Angiography of a patient's head and neck vessel anatomy is indispensable in medicine not only for evaluating vascular-related pathologies like aneurysms, malformations, or atherosclerosis, but also for reliably assessing the vascularization of a particular anatomic region before surgical procedures.

While digital subtraction angiography (DSA) is still considered the gold standard, it has increasingly been replaced by computed tomography angiography (CTA) or magnetic resonance angiography (MRA) during the last years. The main reason for the shift to CTA is the rapid technical evolution in hardware and software, allowing even smaller imaging centers to perform high-quality vessel imaging. During the last decade, CTA came up with substantial progress in terms of accuracy in stenosis^1^ and aneursym detection.^2^ In terms of aneurysm detection, CTA is comparable to DSA and in some instances superior to MRA.^3^ Ultrasound has significant value in carotid artery examinations but features major constraints in terms of intracranial vessel disease.

Applying a standardized CTA procedure, the acquired data are highly reproducible, allowing high-resolution noninvasive vascular imaging. Moreover, CTA is not associated with neurologic complications as with DSA, though the complication rate in DSA is considerably low.^4^

Nonetheless, public awareness regarding the potential long-term risks of ionizing radiation is present.^5^ It has been demonstrated that the applied dose of ionizing radiation correlates linearly with the number of DNA double-strand breaks.^6^ According to the suggested linear no-threshold model, any dose, no matter how small, can induce cancer,^7^ claiming application of ionizing radiation according to the as low as reasonably achievable (ALARA) principle.

Radiation dose is directly proportional to the tube current,^8^ whereas image noise is inversely proportional to the square root of the tube current.^9^ Hence, lowering the tube current proportionally lowers the radiation dose while simultaneously increasing image noise disproportionately.

With consideration of the excellent image quality achievable with state-of-the-art CTA, the question arises to what extent radiation exposure can be reduced by decreasing tube current without risking diagnostic image quality.

The aim of this study was to examine to what extent tube current can be lowered in CTAs of the carotid arteries and the vertebrobasilar system in relation to the established standard protocol. For this purpose, a dedicated algorithm^10,11^ was used to simulate an examination of the identical anatomical region with lower radiation exposure and offering the ability to directly compare the resulting datasets with respect to image quality and noise.

## MATERIAL AND METHODS

### Dose Simulation

There are 2 principle sources for noise in computed tomography (CT) images: quantum noise and electronic noise.^12^ The quantum noise is determined by the number of incident photons collected by the detector. The electronic noise is the result of fluctuation in the electronic components of the data acquisition system.^13^ Electronic noise can be neglected in higher dose scans but becomes important in ultra-low-dose scans. When the number of photons (determining quantum noise) is reduced to the level where the detected signal is as small as the signal from electronic noise, the images will have significantly degraded quality. Photon starvation artifacts occur in ultra-low-dose situations. Therefore, it is desirable to reduce the level of electronic noise in order to improve the image quality in low-dose examinations, which requires the refinement of all electronic components in the x-ray detection system.^13^

Measured x-ray raw data projection represents the object attenuation that can be defined as the ratio of incident source photons and penetrated photons.

In a scan which is reduced in dose by a factor <1 an equally lowered amount of penetrating photons can be expected. In these cases, the increasing noise contribution associated with a lowered number of penetrating photons can be resembled by mathematical methods.

The prototype reconstruction platform ReconCT (version 13.0.0.1, Siemens Healthcare, Forchheim, Germany) is a developmental tool of the manufacturer for algorithm predevelopment with high flexibility. The scanner established processing steps of a standard reconstruction chain (e.g. data processing, weighted filtered backprojection, and image filtering) are built into ReconCT in an identical fashion and only novel contributions, like the noise insertion capabilities, are added as additional features. It allows the reading of raw data and insertion of extra noise based on properly scaled Poisson-distributed random numbers. For this purpose, the quantum noise contribution and an additional scaled normal distributed random number component simulating the electronic noise are taken into account, followed by a subsequent reconstruction of CT images from this data.

To optimize results, a calibration step for quantum noise simulation was performed as follows: a 20-cm water phantom scan was performed in the same scan mode as the clinical data at hand with identical tube voltage and an effective tube current of 200 eff. mAs. This approach ensured the minimization of some systematic effects as detector integration times and numbers of projections per rotation were identical in the calibration step and the clinical data acquisition. From the reconstructed images of the nonmodified raw data, the image noise was determined by placing a 5 cm diameter region of interest (ROI) in the center of the calibration phantom. In an automated iterative process, the noise insertion was repeatedly performed with a target dose level of 6.25% followed by the reconstruction of the lower dose images. Repeated automatic parameter optimization was performed until the image noise reached the expected level.

The electronic noise contribution is a known parameter as the electronic detector components are known and their performance can be easily measured by a scan without x-ray and an analysis of the raw data. Therefore, no calibration was required for this contribution.

All further reconstructions of clinical data were performed based on a calibration set determined by the method described above.

A detailed description of the handling of quantum and electronic noise, tube current modulation, and bowtie filter effects is beyond the scope of this document and can be found in Yu et al.^11^

### Phantom Study

Using a head phantom (3M Germany), consisting of a human skull embedded in plastic, true scans were performed with different dose protocols and compared to the results of simulated dose reductions of the same degree, respectively. Tubes filled with diluted contrast media solution (3% and 4%; 350 mg iodine/mL; Imeron, Bracco Imaging, Konstanz, Germany) were attached to the phantom to simulate contrast-enhanced neck vessels (Figure 1).

**FIGURE 1:**
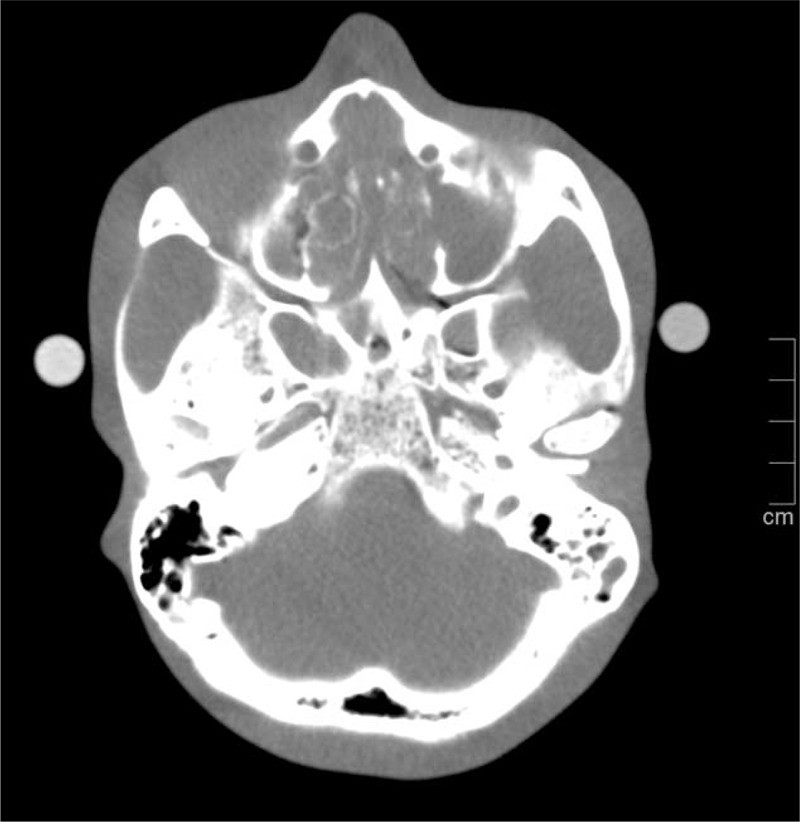
Axial multiplanar reconstruction of a phantom scan at 100 kV and 140 eff. mAs. Tubes filled with diluted contrast media solutions attached to both sides of the phantom were used to simulate contrasted vessels.

Scans were performed on a 128-section CT system (Definition AS+; Siemens Healthcare) with a collimation of 128 × 0.6 mm and a pitch of 0.6. To resemble the parameters the automatic kV selection chose in the patient study (see below), a tube voltage of 100 kV at 160, 120, 80, and 40 eff. mAs was selected. The raw data were transferred to a dedicated workstation, and images of the different tube currents were reconstructed using a soft tissue kernel (B31f), a field of view of 180 mm, a slice thickness of 0.75 mm, and an increment of 0.5 mm.

Additionally, dose simulations of 75%, 50% and 25% were derived from the 160 eff. mAs dataset, resembling the true scans at 120, 80, and 40 eff. mAs.

Image analysis was performed on a standard workstation (syngo.via, VA30, Siemens Healthcare, Erlangen, Germany) in 3D multiplanar mode (slab thickness 1 mm, window width 700 Hounsfield unit (HU), and window center 100 HU). In each of the true scans, 10 ROIs were placed within the contrast media-filled tubes (5 ROIs/3% and 4% dilution) at the level of the skull base. These ROIs were duplicated to the matched 75%, 50%, and 25% datasets. Mean attenuation (HU) and standard deviation (HU) were noted, and standard deviation was considered as a measure of noise.

### Patient Study

A total of 30 patients (7 females and 23 males) with a mean age of 68 years (range: 49–85 years) scheduled for CTA of the supra-aortic vessels were consecutively included in this prospective study. All patients had the suspicion of stenosis of the carotid arteries. Of these, 14 patients had a history of prior neck surgery. All studies were conducted in accordance with the guidelines of the Declaration of Helsinki and in coordination with institutional board guidelines of the University of Erlangen-Nuremberg. Written informed consent was obtained from each patient.

### Computed Tomography Angiography/Scanning Protocol

The CTA scans were performed on the same CT system as the phantom measurements. The scan range included the lateral ventricles to the aortic arch. Scanner settings were as follows: Collimation 128 × 0.6 mm, pitch 0.6, CareDose4D on, Care kV on, Ref. 120 kV, Qual Ref. mAs 140.

Circulation time was individually calculated by using a test bolus injection with 10 mL contrast media solution at a flow rate of 4 mL/s, chased by a 40-mL saline bolus at the same flow rate. The ROI to determine the bolus arrival time (BAT) was placed in the aortic arch. The individual start delay of the diagnostic scan was calculated by using the formula BAT + 2 seconds. Fifty milliliters of contrast media solution followed by a 40-mL saline flush were injected at the same flow rate of 4 mL/s for the diagnostic scans. Tube voltage (kV) and current (mAs), CT dose index volume (CTDIvol, milligray [mGy]), and dose-length product (DLP, mGy ×cm) were recorded from the patient protocol for the diagnostic scans.

The raw data were transferred to a dedicated dose simulation workstation, and anonymized images were reconstructed at a level of 100%, 75%, 50% and 25% of the initial dose using a soft tissue kernel (B31f), a field of view of 180 mm, a slice thickness of 0.75 mm, and an increment of 0.5 mm.

### Objective Image Analysis

Objective image analysis was performed on a standard workstation (see above) in 3D multiplanar mode (slab thickness 1 mm, window width 700 HU, and window center 100 HU). One radiology fellow (SE; 2 years of experience in neurovascular imaging) placed ROIs centrally within the vessel lumen on axial images, covering about two-thirds of the cross-sectional area. This was done in the right-sided common carotid arteries at the level of the shoulders, cranial the shoulders, in the carotid bifurcation, and in the lacerum (C3) segment of the internal carotid artery in the 100% dose images. These ROIs were replicated in the matched 75%, 50%, and 25% datasets by copy and paste. The workstation then automatically provided data for this specific ROI. Mean attenuation (HU) and standard deviation (HU) were noted. Standard deviation was considered as a measure for image noise. Signal-to-noise ratio (SNR) was calculated by dividing the mean attenuation of the ROIs by their standard deviation.

### Subjective Image Analysis

Two board certified radiologists (MK and ML; 7 and 17 years of experience in neurovascular imaging, respectively) evaluated the images blinded regarding the patient name and the degree of dose simulation in random order in separate sessions in consensus. The datasets were read interactively on a standard workstation with syngo.via in 3D multiplanar mode (slab thickness 1 mm, window width 700 HU, and window center 100 HU). Individual adjustment of window width and center was allowed. A quality score was assigned to the following vessel segments: brachiocephalic trunk, common carotid artery, carotid bifurcation, external carotid artery segments (main stem, superior thyroid artery, lingual artery, facial artery, maxillary artery, and superficial temporal artery), internal carotid artery segments (C1–C7),^14^ vertebral artery segments (V1–V4), and basilar artery (n = 40). All segments were analyzed in each dataset, excluding those segments that were occluded or ligated in prior rescection.

The following scoring system was used: 1 = no diagnostic vessel definition; 2 = poor vessel definition—diagnostic confidence significantly reduced; 3 = moderate vessel definition—sufficient for diagnosis; 4 = good vessel definition; and 5 = excellent vessel definition. Additionally, the segments were dichotomized into nondiagnostic (no diagnostic or poor vessel definition) and diagnostic (moderate, good, and excellent vessel definition). Ten CTA studies were reevaluated 1 month later independently by readers 1 and 2 to determine interobserver agreement with the suggested scoring system.

### Statistical Analysis

In the phantom study, a Mann–Whitney *U* test was used to compare the mean attenuation of the entity of true scans at different dose levels with the mean attenuation of the simulated scans. Sign test were used to compare the standard deviations of the ROIs as a measure of noise between the true scans at 100 kV/160, 120, 80, and 40 eff. mAs and the matched simulations.

In the patient study, sign tests were used to compare segmental quality scores of the subjective analysis and to compare standard deviation and SNR of the objective image analysis. Interobserver agreement for subjective image analysis was determined by Cohen κ statistics (no agreement, κ = 0; slight agreement, κ = 0.1–0.2; fair agreement, κ = 0.21–0.4; moderate agreement, κ = 0.41–0.6; substantial agreement, κ = 0.61–0.8; and almost perfect agreement, κ = 0.81–1).

In all statistical calculations, *P* values ≤ .05 were considered statistically significant. All calculations were done using SPSS for Windows 20.0 (IBM, Armonk, NY).

## RESULTS

### Phantom Study

Three to four percent of contrast media-filled tubes resembled contrast-enhanced vessels with an average attenuation of 330.0 ± 46.0 HU in the true scans (n = 40). Comparing the mean attenuation of phantom vessels yielded no significant differences between the simulated scans with 75%, 50%, and 25% of the original dose and the true scans with the same degree of radiation dose reduction (*P* = .111 over all groups, n = 30).

The difference in mean attenuation between the true and the simulated scans was not significant (*P* = .65). While mean attenuation in vessel phantoms was not significantly altered by radiation dose reduction in simulations and true scans, the noise measured as standard deviations in ROIs increased along with radiation dose reduction.

Comparing the noise differences between true and simulated scans of vessel phantoms, we found no significant noise differences between true scans and simulated scans at all radiation dose levels (*P* = .59 for 75%, *P* = .81 for 50%, *P* = .86 for 25%; see Figure 2). All differences in noise levels between the different radiation dose reduction levels were significant (all *P* < .028).

**FIGURE 2:**
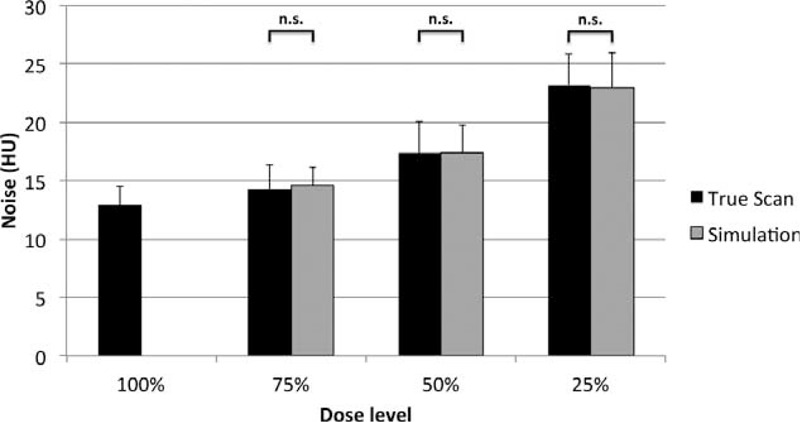
Dose reduction increases noise. Comparing the simulated levels of noise with the measured noise at dose-reduced true scans revealed no significant differences (n.s.) between the noise of true (black columns) and simulated scans (gray columns) at dose reduction levels of 75%, 50% and 25% of the original dose.

### Patient Study

All 30 CTAs were performed at 100 kV using automated kV selection based on the attenuation values of the scout views. Mean tube current was 166.0 mAs (range: 119–219 mAs), mean CTDIvol (for a 32-cm CTDI phantom) was 6.55 ± 0.81 mGy (range: 4.71–8.63 mGy), and mean DLP was 211.23 ± 34.5 mGy cm (range: 142–265 mGy cm).

### Objective Image Analysis

Mean attenuation, standard deviation, and SNR of the ROIs in the right-sided common carotid arteries at the level of the shoulders, cranial the shoulders, in the carotid bifurcation, and in the lacerum (C3) segment of the internal carotid artery are shown in Table 1. Significant differences were detected for the comparisons of standard deviation (all *P* < .001) and SNR (all *P* < .001) of all dose groups.

**TABLE 1:**
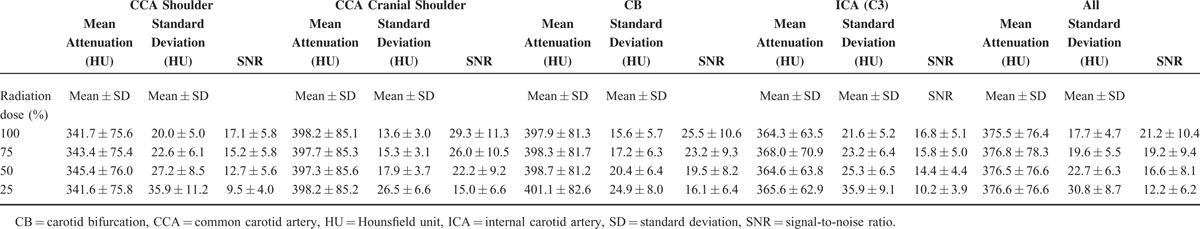
Mean Attenuation, Standard Deviation, and SNR in the Common Carotid Arteries at the Level of the Shoulders, Cranial the Shoulders, in the Carotid Bifurcation, in the Lacerum (C3) Segment of the Internal Carotid Artery, and in All of These Segments for All Patients in the 100% Radiation Dose Reconstructions and the 3 Dose Simulations of 75%, 50% and 25% of the Original Dose

### Subjective Image Analysis

Among 4800 vessel segments (40 different vessel segments × 30 patients × 4 dose simulations), 4664 segments were scored and 136 segments had to be excluded from evaluation because of occlusion or prior resection. The quality scores, including percentiles, median, diagnostic, and nondiagnostic segments, are shown in Table 2 (for a visual impression, see Figure 3). The quality scores of the 3 lower dose simulation groups (75%, 50%, and 25% radiation dose) differed statistically significant from the 100% dose datasets (all *P* < 0.001). After dichotomization into the categories of diagnostic and nondiagnostic image quality, this significant difference vanished for the comparison of the 100% and 75% dose datasets (*P* = .50) and remained for the comparison with the 50% and 25% dose groups (*P* < .001). The scoring system proved to be reproducible with substantial interobserver agreement (κ = .64).

**TABLE 2:**
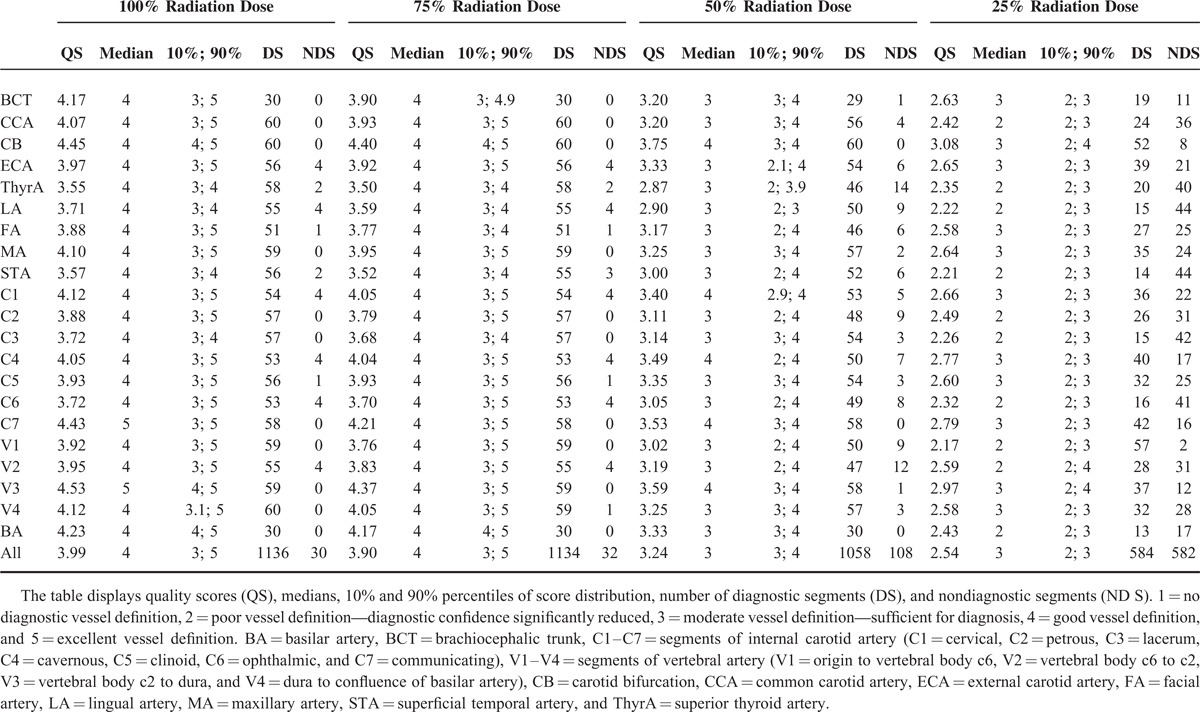
Subjective Quality Scores for All Patients in the 100% Dose Reconstructions and the 3 Dose Simulations of 75%, 50% and 25% of the Original Radiation Dose

**FIGURE 3:**
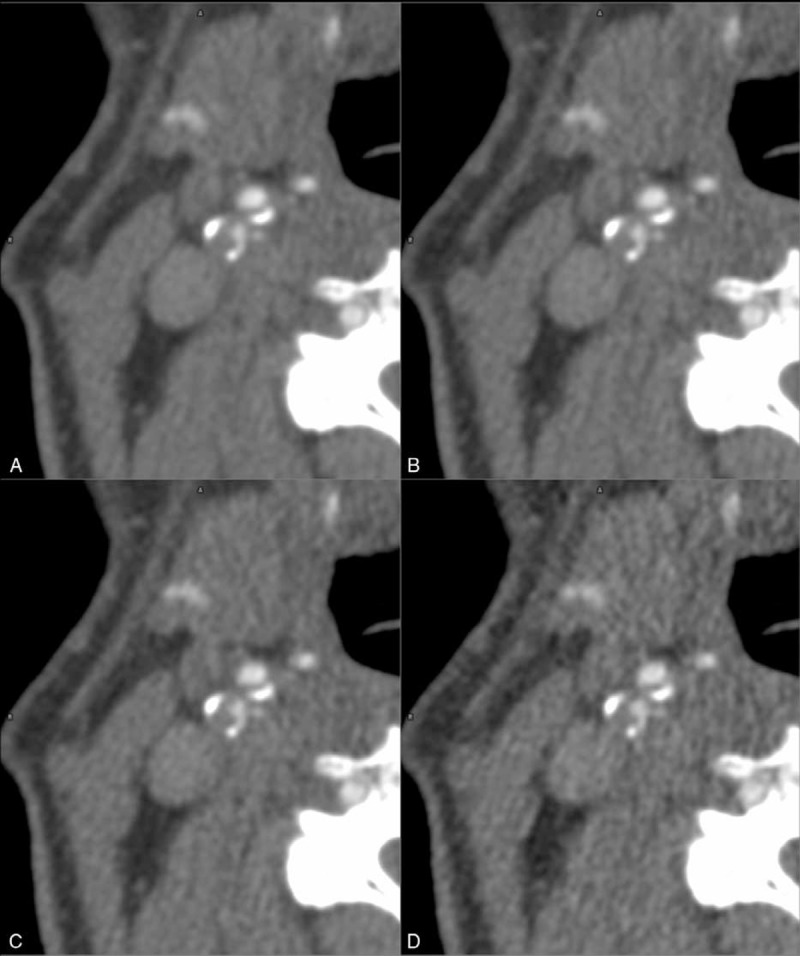
Dose reduction increases noise. Comparing an axial magnified 100% true scan of a right-sided proximal internal carotid artery stenosis (A) with simulated scans at 75% (B) 50% (C), and 25% (D) of the original dose reveals the increase of noise accompanying dose reduction.

## DISCUSSION

Radiation dose reduction in CT has attracted increasing interest during the past years.^5^ Several efforts have been taken to bypass the problem of lower dose scans primarily resulting in reduced image quality by, for example, automatically adapting tube currents and voltages or performing iterative reconstructions.^15–18^

In CTAs, methodical problems arise from the need of exact contrast media bolus timing. Optimum contrast is guaranteed by exact timing, which is either managed by determining the delay before initiation of the scan using a test bolus or by triggering the initiation by bolus tracking.^19^ If an investigator wanted to perform several scans in a row, the comparability of scans acquired in a serial manner would be seriously limited due to different—and in most cases suboptimal—contrast phases.

Besides these methodical concerns, scanning a patient with different radiation dose protocols in a serial manner is not justifiable under ethical considerations, as the cumulated dose applied to any given patient in such a study would result in a multiple of the dose actually needed to perform sufficient diagnostics. Reducing the tube current arbitrariely would pose the risk of non-diagnostic results, which is not tolerable because of contrast material and radiation exposure.

For these reasons, the determination of the point at which the extent of radiation dose reduction and resulting image quality are in best balance remains complicated.

In 1997, Mayo et al^20^ suggested adding noise to CT images to simulate scans with lower tube current and consecutively lower radiation dose. By applying a modified algorithm, we were able to simulate CTAs of the supra-aortic arch and neck vessels with lower tube current and directly compare original data with data acquired under simulated radiation dose reduction.

To verify the algorithm of noise simulation, we performed phantom scans. As shown by true and simulated phantom scans at different radiation dose reduction levels, we proved our algorithm to perform well in simulating radiation dose reductions to 25% of the original dose, which comprises a resulting tube current of 40 mAs.

In the patient study, all diagnostic scans were performed at a radiation dose of 100% of our standard protocol for the ethical and methodical reasons described above. In the objective image analysis, ROIs were placed in different levels of the right-sided carotid arteries. Determining the noise level by measuring the standard deviation within a ROI is an auxiliary construct. In an idealized situation, the standard deviation within a ROI is solely determined by quantum noise and is inversely proportional to the square root of the tube current and thus radiation dose. In this ideal situation, reducing the tube current to 25% doubles the image noise. In reality, however, other components additionally influence the standard deviation of a ROI to a more or less extent, for example streak artifacts. These components might carry disproportionately high weight in certain areas, for example at the skull base, shoulder level, or in vessels with higher grade calcifications. The common carotid artery above the shoulders is superimposed by only few artifacts. This results in the lowest noise at the radiation dose level of 100% (13.6 ± 3.0 HU; see Table 1), and therefore the highest SNR. Only in this segment, the noise measured within a ROI is inversely proportional to the square root of the radiation dose, as the standard deviation nearly doubles at a radiation dose level of 25% (26.5 ± 6.6 HU) compared to the 100% level (13.6 ± 3.0 HU). In the other segments, this relationship is weakened, as actual conditions such as wall calcifications, skull base artifacts, and so on lead to a per se higher standard deviation at the 100% radiation dose level. In these segments, the artifact-derived noise contributes in a nonnegligible extent to the standard deviation. For this reason, the SNR in these segments is reduced compared to the relatively artifact-free common carotid artery above the shoulders.

The noise increased along with simulated lower tube current in a significant manner. This increase of noise was visible during the subjective image analysis. Increased noise led to the subjective impression of decreased image quality in a significant extent between all simulated reduction datasets in comparison to the 100% true scan. Nonetheless, decreased subjective image quality not necessarily results in decreased diagnostic accuracy, proven by the nonsignificant differences of the 100% true scan in comparison with the 75% simulation in regards of diagnostic image quality.

Ultrasound and MRA are alternatives for evaluation of vessel pathologies without exposing the patient to radiation at all. Nonetheless, ultrasound features limitations in terms of distinguishing subtotal from total occlusion, artifacts due to calcifications, high operator variability, and detection of intracranial lesions.^21^ CTA in contrast offers optimal reproducibility.^22^

MRA can be performed without contrast agent or in a contrast-enhanced manner with different sequences, with each one suffering from a different combination of drawbacks including overestimation of stenosis degree, artifacts, long acquisition times, and constricted volume coverage.^21,23–28^

One limitation of this work is its adaption to the standard protocol of our institute for CTA of the supra-aortic arch and neck vessels (140 Qual Ref. mAs and 120 kV with automatic kV selection, resulting in a reduced tube voltage of 100 kV in all patients). Nonetheless, the principle of simulating tube current reduction by artificially adding noise can easily be applied to different protocols. Our study, moreover, did not include iterative reconstructions, as these iterative processes would have interfered with the simulated noise, making it difficult to sufficiently assign observed effects to the opposed effects of noising and de-noising.

Performing diagnostic CTAs with reduced tube current might not be applicable or warrantable in every single patient just as a matter of principle. But performing low-dose imaging can be of great interest whenever scanning younger patients or scanning patients several times during a given period, for example in cases of follow-ups or control scans. With an already known lesion that solely needs to be followed up, all these control scans in our eyes could be performed with reduced tube current to decrease the patient's cumulative radiation dose.

Although it could be argued that standard protocols already provide relatively low effective radiation doses and x-ray exposure may be less of an issue in a predominantly high-aged patient group, the ALARA principle should still be followed.

As stated above, one major drawback of CTA is its radiation exposure. This study therefore suggests an easy-to-implement method of simulating CTAs of the carotid arteries and the vertebrobasilar system vessels with lower tube current for radiation dose reduction by artificially adding noise. We moreover provide evidence that lowering the radiation dose in a moderate extent to 75% of the standard dose does not significantly alter diagnostic image quality. To the best of our knowledge, this is the first study investigating the effectiveness of scan simulations in determining the extent of reasonable radiation dose reduction in CTAs of the carotid arteries and the vertebrobasilar system vessels.

## References

[1] Kim JJ Dillon WP Glastonbury CM Sixty-four-section multidetector CT angiography of carotid arteries: a systematic analysis of image quality and artifacts. *AJNR Am J Neuroradiol* 2010; 31:91–99. 19729539 10.3174/ajnr.A1768 PMC7964077

[2] Li Q Lv F Yao G 64-section multidetector CT angiography for evaluation of intracranial aneurysms: comparison with 3D rotational angiography. *Acta Radiol* 2014; 55:840–846. 24065201 10.1177/0284185113506138

[3] Kouskouras C Charitanti A Giavroglou C Intracranial aneurysms: evaluation using CTA and MRA. Correlation with DSA and intraoperative findings. *Neuroradiology* 2004; 46:842–850. 15448952 10.1007/s00234-004-1259-2

[4] Willinsky RA Taylor SM TerBrugge K Neurologic complications of cerebral angiography: prospective analysis of 2,899 procedures and review of the literature. *Radiology* 2003; 227:522–528. 12637677 10.1148/radiol.2272012071

[5] Barrett B Stiles M Patterson J Radiation risks: critical analysis and commentary. *Prev Med* 2012; 54:280–282. 22227282 10.1016/j.ypmed.2011.12.017

[6] Kuefner MA Grudzenski S Schwab SA DNA double-strand breaks and their repair in blood lymphocytes of patients undergoing angiographic procedures. *Invest Radiol* 2009; 44:440–446. 19448553 10.1097/RLI.0b013e3181a654a5

[7] Shah DJ Sachs RK Wilson DJ Radiation-induced cancer: a modern view. *Br J Radiol* 2012; 85:e1166–1173. 23175483 10.1259/bjr/25026140 PMC3611719

[8] Frush DP Donnelly LF Rosen NS Computed tomography and radiation risks: what pediatric health care providers should know. *Pediatrics* 2003; 112:951–957. 14523191 10.1542/peds.112.4.951

[9] Solomon JB Li X Samei E Relating noise to image quality indicators in CT examinations with tube current modulation. *AJR Am J Roentgenol* 2013; 200:592–600. 23436849 10.2214/AJR.12.8580

[10] Frush DP Slack CC Hollingsworth CL Computer-simulated radiation dose reduction for abdominal multidetector CT of pediatric patients. *AJR Am J Roentgenol* 2002; 179:1107–1113. 12388482 10.2214/ajr.179.5.1791107

[11] Yu L Shiung M Jondal D Development and validation of a practical lower-dose-simulation tool for optimizing computed tomography scan protocols. *J Comput Assist Tomogr* 2012; 36:477–487. 22805680 10.1097/RCT.0b013e318258e891

[12] Zhao Z Gang GJ Siewerdsen JH Noise, sampling, and the number of projections in cone-beam CT with a flat-panel detector. *Med Phys* 2014; 41:061909. 24877820 10.1118/1.4875688 PMC4032401

[13] Yu L Liu X Leng S Radiation dose reduction in computed tomography: techniques and future perspective. *Imaging Med* 2009; 1:65–84. 22308169 10.2217/iim.09.5 PMC3271708

[14] Bouthillier A van Loveren HR Keller JT Segments of the internal carotid artery: a new classification. *Neurosurgery* 1996; 38:425–432. 8837792 10.1097/00006123-199603000-00001

[15] Fuentes-Orrego JM Sahani DV Low-dose CT in clinical diagnostics. *Expert Opin Med Diagn* 2013; 7:501–510. 23941284 10.1517/17530059.2013.826647

[16] Morsbach F Frauenfelder T Alkadhi H Modern techniques for dose reduction in computed tomography imaging. *Praxis* 2013; 102:865–868. 23823685 10.1024/1661-8157/a001352

[17] McCollough CH Primak AN Braun N Strategies for reducing radiation dose in CT. *Radiol Clin North Am* 2009; 47:27–40. 19195532 10.1016/j.rcl.2008.10.006 PMC2743386

[18] Eller A Wuest W Kramer M Carotid CTA: radiation exposure and image quality with the use of attenuation-based, automated kilovolt selection. *AJNR Am J Neuroradiol* 2014; 35:237–241. 23907241 10.3174/ajnr.A3659 PMC7965763

[19] Rodrigues JC Mathias H Negus IS Intravenous contrast medium administration at 128 multidetector row CT pulmonary angiography: bolus tracking versus test bolus and the implications for diagnostic quality and effective dose. *Clin Radiol* 2012; 67:1053–1060. 22520034 10.1016/j.crad.2012.02.010

[20] Mayo JR Whittall KP Leung AN Simulated dose reduction in conventional chest CT: validation study. *Radiology* 1997; 202:453–457. 9015073 10.1148/radiology.202.2.9015073

[21] Saba L Anzidei M Sanfilippo R Imaging of the carotid artery. *Atherosclerosis* 2012; 220:294–309. 21968317 10.1016/j.atherosclerosis.2011.08.048

[22] Saba L Sanfilippo R Pascalis L Carotid artery wall thickness and ischemic symptoms: evaluation using multi-detector-row CT angiography. *Eur Radiol* 2008; 18:1962–1971. 18404269 10.1007/s00330-008-0962-5

[23] Anzalone N Scomazzoni F Castellano R Carotid artery stenosis: intraindividual correlations of 3D time-of-flight MR angiography, contrast-enhanced MR angiography, conventional DSA, and rotational angiography for detection and grading. *Radiology* 2005; 236:204–213. 15955853 10.1148/radiol.2361032048

[24] Hartung MP Grist TM Francois CJ Magnetic resonance angiography: current status and future directions. *J Cardiovasc Magn Reson* 2011; 13:19. 21388544 10.1186/1532-429X-13-19 PMC3060856

[25] Kramer H Runge VM Morelli JN Magnetic resonance angiography of the carotid arteries: comparison of unenhanced and contrast enhanced techniques. *Eur Radiol* 2011; 21:1667–1676. 21476128 10.1007/s00330-011-2110-x

[26] Lell M Fellner C Baum U Evaluation of carotid artery stenosis with multisection CT and MR imaging: influence of imaging modality and postprocessing. *AJNR Am J Neuroradiol* 2007; 28:104–110. 17213434 PMC8134121

[27] Nederkoorn PJ Mali WP Eikelboom BC Preoperative diagnosis of carotid artery stenosis: accuracy of noninvasive testing. *Stroke* 2002; 33:2003–2008. 12154253 10.1161/01.str.0000021900.58396.44

[28] Raoult H Gauvrit JY Schmitt P Non-ECG-gated unenhanced MRA of the carotids: optimization and clinical feasibility. *Eur Radiol* 2013; 23:3020–3028. 23783783 10.1007/s00330-013-2931-x

